# Intravascular Crawling of Patrolling Monocytes: A Lèvy-Like Motility for Unique Search Functions?

**DOI:** 10.3389/fimmu.2021.730835

**Published:** 2021-09-17

**Authors:** Rocío Moreno-Cañadas, Laura Luque-Martín, Alicia G. Arroyo

**Affiliations:** Molecular Biomedicine Department, Centro de Investigaciones Biológicas Margarita Salas (CIB-CSIC), Madrid, Spain

**Keywords:** patrolling monocytes, crawling, search theory, Lèvy-like walk, intravascular surveillance, microparticle deposits, αMβ2 integrin, CD36

## Abstract

Patrolling monocytes (PMo) are the organism’s preeminent intravascular guardians by their continuous search of damaged endothelial cells and harmful microparticles for their removal and to restore homeostasis. This surveillance is accomplished by PMo crawling on the apical side of the endothelium through regulated interactions of integrins and chemokine receptors with their endothelial ligands. We propose that the search mode governs the intravascular motility of PMo *in vivo* in a similar way to T cells looking for antigen in tissues. Signs of damage to the luminal side of the endothelium (local death, oxidized LDL, amyloid deposits, tumor cells, pathogens, abnormal red cells, etc.) will change the diffusive random towards a Lèvy-like crawling enhancing their recognition and clearance by PMo damage receptors as the integrin αMβ2 and CD36. This new perspective can help identify new actors to promote unique PMo intravascular actions aimed at maintaining endothelial fitness and combating harmful microparticles involved in diseases as lung metastasis, Alzheimer’s angiopathy, vaso-occlusive disorders, and sepsis.

## Introduction

Among the two main subsets of circulating monocytes, non-classical monocytes (CCR2^-^ CX3CR1^high^Ly6C^low^ in mouse, CCR2^-^ CX3CR1^high^CD14^dim^CD16^+^ in humans) are also called patrolling monocytes (PMo) by their ability to actively patrol the vascular endothelium to search for harmful microparticles (pathogens, circulating tumor cells, amyloid deposits, abnormal red blood cells, etc.) or dying endothelial cells and promote their removal to restore homeostasis (1–5). Therefore, PMo are considered protective in pathological contexts such as lung metastasis, Alzheimer’s disease angiopathy, atherosclerosis, sepsis, and vaso-occlusive disorders (1–3, 6–9). Once PMo extravasate, although they do it rarely, their actions can be beneficial or detrimental depending on the context and the environmental cues that drive their differentiation into distinctive types of macrophages (10–13).

Since pioneering studies by Geissmann’s group (1), PMo have been observed patrolling in the microvasculature of dermis, mesentery, brain, lung, kidney and muscle, and in carotid and femoral arteries under homeostatic and inflammatory conditions [reviewed in (4)]. PMo differentiate from classical monocytes in defined vascular niches of the bone marrow and spleen through an intermediate subpopulation (5, 14). PMo numbers in the circulation are also regulated by β-adrenergic stimulation during exercise and stress (15, 16), pattern recognition receptors as NOD2 (17), soluble factors as tumor exosome-derived PEDF (18), and chemokines as CX3CL1 (17, 19, 20) indicating that their abundance is exquisitely sensitive to signals triggered by damage, stress or inflammation as a protective response.

PMo perform their surveillance function by crawling on the endothelium (1, 19), but the influence of their primary PMo search function into their motility has been overlooked. Recent reports suggest, however, that particle encounter and patrolling activity can be related (21, 22). Following the “search theory,” we propose to consider PMo crawling as a movement guided by the “exploration-exploitation trade off” (23), which comprises, but is not limited to, non-informed explorative search without much guidance cues and informed exploitative search with input from the environment. We will describe intravascular PMo crawling from this perspective taking as a reference walks described for T cells in search of an antigen in the lymph node and other tissues (23, 24).

## Patrolling Monocyte Crawling: A Search Mode Motility

Intravascular PMo crawling is defined as the scanning movement of the apical surface of endothelial cells, which in the microvasculature does not depend on the direction of blood flow, travels long distances without greater directionality, performs looped trajectories and without immediate extravasation (1, 4, 25). In large vessels, PMo crawling displays an overall with-the-flow direction with no typical hairpin and loop patterns (26). Unlike rolling leukocytes, PMo adhere firmly while crawling and are slower by a factor of 100 to 1,000 (1).

As an exploration movement, intravascular PMo crawling must transition among random motility modes balancing migration speed with sufficient dwell time and meandering for a thorough survey of the endothelial surface. Diffusive random crawling involves walks with little or no directional persistence (Brownian-type tracks) with the intention of surveying the largest surface in the shortest possible time to find local alarm signals. Tracks of PMo crawling consistent with this mode are observed in steady-state and inflammatory conditions (1, 2, 25–28). However, PMo modify their crawling pattern in the presence of local endothelial damage (19, 29) or microparticles (8, 21, 26). Tracks in these cases resemble superdiffusive random walks, particularly the Lèvy-like walk, which consists of an alternation of long, quick, and directed trajectories (flights) with short and slow random turning directions. In this situation, PMo no longer perform only exploration, but signal-informed movement to find their final target. Both diffusive random and Lèvy-like crawling can coexist in the microvasculature [see Movie S2 in (1)]. Additionally, a high density of local damage will disrupt PMo Lèvy-like walk and promote confined crawling by the frequent encounter of PMo with their target. Intravascular crawling often ends with PMo detachment and continuation of patrol. PMo rarely perform a truly directional crawling leading to transendothelial migration, in contrast to classical and intermediate monocytes that arrest and transmigrate more frequently (13, 27), so we will not consider it further as it is not related to intravascular surveillance.

Kinetic parameters of the different types of intravascular PMo search crawling are summarized in **Table 1**. In general, diffusive random PMo crawling is faster and longer to scan large surfaces efficiently. In contrast, Lèvy-like PMo walks comprise slower and shorter tracks connected by fast-speed steps (although information on individual tracks of this type is not available). These parameters are further reduced during confined crawling. Speed ​​better captures the different modes, while straightness seems less informative in describing PMo crawling, as both random and confined walks can show similar values, however reflecting different search and find behaviors (**Table 1**). Analogous motilities are found in NKT cells randomly searching for antigens in liver sinusoids (31) or T cells performing Lèvy-like and confined walks to look for antigen in tissues (30, 32, 33, 35) (**Table 1**).

**Table 1:** Cellular kinetic parameters of the proposed crawling modes in various territories and conditions: mean speed (μm/min), length (μm), duration (min), and straightness (distance traveled/length of the trajectory).

Diffusive random crawling		Lèvy-like walk		Confined crawling		References
PMo	NKT cells	PMo	LT	PMo	LT	
**Lung (capillaries)**						
*Healthy* ≈10 μm/min		*Tumor cells (4 h)* 6.7 μm/min	*LPS lung* 2.3 μm/min	*Tumor cells (24 h)* 1.5 μm/min	*LPS lung* 0–1 μm/min	(3, 30)
**Other organs**						
*Healthy* Kidney ≈9 μm/min ≈80 μm ≈9 min ≈0.6	*Healthy* Liver sinusoids 16.5 μm/min 0.4	*TLR7/8 agonist* Kidney ≈7.5 μm/min ≈150 μm ≈22 min ≈0.3	*Tumor skin* 4.3 μm/min 0.4 *Infected brain* 6.4 μm/min		*Tumor skin* 1.4 μm/min 0.5	(2, 31–33)
**Arteries**						
*Healthy* Carotid 36 μm/min 134 μm 4.7 min 0.2 Femoral 12 μm/min nd ≈0.6		*Hyperlipidemia* Carotid 30 μm/min 140 μm 6.1 min 0.22 Femoral 5 μm/min ≈200 μm ≈0.6 *TLR7/8 agonist* Carotid 19 μm/min 124 μm 5.7 min 0.1		*Atheroma plaque* Carotid 20 μm/min 167 μm 7.7 min 0.05		(21, 26)
**Venules**						
*Healthy* Mesentery ≈9 μm/min ≈200 μm ≈20 min ≈0.6 Dermis 17 μm/min 249 μm 14 min 0.6 Cremaster ≈10 μm/min 147.3 μm ≈0.7		*TLR7/8 agonist* Mesentery ≈5-6 μm/min ≈180 μm ≈23 min ≈0.4				(1, 27, 29, 34)

Most of the values ​​given are approximate; for accurate values, please refer to the original articles. The parameters for PMo and NKT are intravascular, while for LT they are in the tissue. Note that the parameters do not correspond to individual tracks but to the average of all observed tracks.

PMo, patrolling monocytes; NKT cells, natural killer T cells; LT, T lymphocytes.

**Figure 1:**
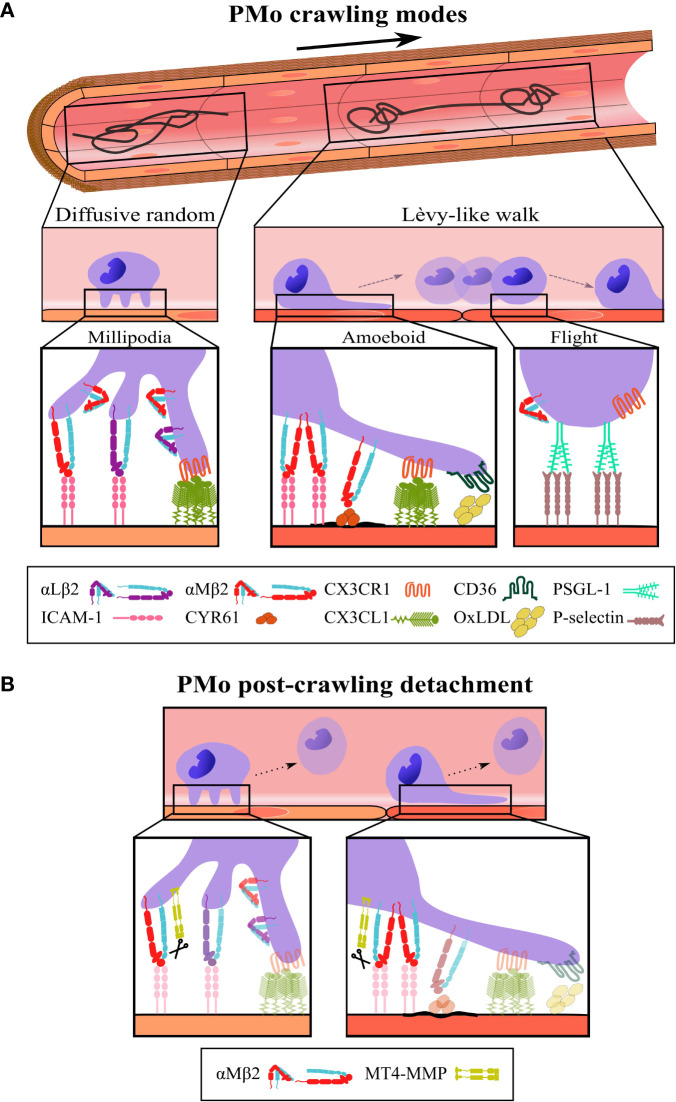
Schematic representation of the different modes, steps, and players in intravascular PMo crawling. (**A**, Top) Representative tracks performed by PMo crawling on the vascular endothelium according to the search theory: diffusive random crawling (left) and Lèvy-like walk (right); the bold arrow indicates blood flow direction. Middle, side views of PMo morphology (round and elongated) and the corresponding locomotion modes (millipodia and amoeboid) during diffusive random crawling and Lèvy-like walks; in the last, the flight phase is also indicated. Bottom, magnifications display the molecular players and interactions relevant to each type and step of crawling as described in the text; molecular interactions depicted for the flight phase are speculative. **(B)** The cleavage of αM integrin by the protease MT4-MMP is proposed as a possible mechanism for PMo post-crawling detachment (12), an important step to maintain PMo intravascular surveillance; scissors indicate cleavage. The β2 integrins are represented in the folded (inactive) and extended (active) conformations.

The morpho-dynamics and the locomotion mode of PMo are not well defined. PMo appear round and seem to crawl *in vivo* in a millipodia-like manner (36) during diffusive random crawling (2, 28). In this mode, cells do not polarize, probably allowing them to move faster. During Lèvy-like crawling, PMo alternate between elongating, while crawling in a more meticulous manner by an amoeboid movement, and being round during flights to the next location (3, 8, 21, 29) (**Figure 1A**). The possible mechanisms underlying the amoeboid locomotion of PMo (actin polymerization, blebbing, etc.) remain unexplored (37).

## Regulators of Diffusive Random *Versus* Lèvy-Like PMo Crawling

We will review recognized intrinsic and extrinsic players in PMo crawling from the search theory perspective and suggest how they can determine diffusive random and Lèvy-type crawling modes. Particularly, we will highlight the relevance of local endothelial damage signals in PMo locomotion.

### Diffusive Random Crawling

PMo perform diffusive random crawling in most steady-state and inflammatory contexts to explore large endothelial areas without expecting much damage. These kinds of tracks are observed in PMo crawling in mesentery vessels and in arteries but also in dermis, lung, and kidney capillaries (1–3, 21, 25–29). For this type of crawling, PMo need to be sufficiently attached to resist shear stress but with dynamic adhesions to allow fast movement. In the microvasculature, PMo adherence depends on β2 integrins, mostly on αLβ2 integrin (LFA-1, CD11a/CD18) in steady-state conditions by its interaction with ICAM-1 and with additional contribution of αMβ2 integrin (Mac-1, CD11b/CD18) in inflamed conditions (1, 2, 27, 38) (**Figure 1A**). Indeed, αMβ2 integrin seems to determine the fast velocity of diffusive random crawling in steady-state conditions since its inhibition does not affect the abundance of PMo crawlers, but it reduces their speed in mesenteric vessels (29). Diffusive random crawling is also favored in the microvasculature by the interaction of CX3CR1 in PMo with CX3CL1 (**Figure 1A**), a transmembrane ligand abundantly produced by endothelial cells in lung and kidney (1, 3, 29, 38). Moreover, in vascular territories with high shear stress like arteries and the glomerulus, resistance to detachment to support diffusive random crawling is provided by the interaction of α4β1 integrin with its endothelial ligand VCAM-1, with minor or no role of CX3CR1 (26, 38), probably related to CX3CL1 downregulation by shear stress (39).

So far millipede-like crawling has been described in T cells in which it relied on the rapid turnover of traction points formed by high-affinity αLβ2 integrin interactions with endothelial ICAM-1 (36). However, since this type of T cell motility leads to extravasation and not to intraluminal surveillance as in PMo, further research will be required to explore if a similar β2 integrin-mediated mechanism accounts for PMo millipodia-based crawling (40). Nevertheless, the drastic reduction of this type of crawling in arteries in steady state in the absence of kindlin-3, an inside-out regulator of β2 integrins, points to the requirement of high-affinity β2 integrin interactions (22). Accordingly, CX3CR1 favors diffuse random crawling of PMo, probably by its outside-in upregulation of β2 integrin affinity (41) as also supported by reduced PMo crawling in inflammatory conditions under GPCR signaling inhibition (2, 29). Of note, endothelial ligands involved in diffusive random PMo crawling are not distributed homogeneously on the luminal side of the endothelial cells increasing the possibility that they serve as footholds for the millipodia. Thus, apical microdomains organized by tetraspanins or the actin cytoskeleton contain pre-formed clusters of about 2.5 ICAM-1 (42) and 3-to-7 CX3CL1 (43, 44) molecules (**Figure 1A**) and the matricellular protein CYR61/CCN1, another αMβ2 integrin ligand, also forms hotspots in the mesenteric vessels (29). Interestingly, the nanoarchitecture of the apical endothelial membrane is sensitive to factors such as shear stress or the cytokine TNFα, able to promote upward protrusions of about 160 nm, that increase the abundance and/or accessibility of ICAM-1 nanoclusters (45, 46). These effects could influence PMo intravascular crawling.

### Lèvy-Like Crawling

How do PMo perceive that they have to increase the frequency of Lèvy-like walks for a more efficient search, especially for small targets, and how do they re-adapt their arsenal of adhesion and chemokine receptors to this type of movement?

PMo show an amoeboid morphology when crawling in the slow phase of Lèvy-like walk suggestive of signals driven by β2 integrin interactions as shown in neutrophils (47, 48). This is supported by the presence of a few Lèvy-like tracks in steady-state in the lung and kidney capillaries, territories with abundant endothelial CX3CL1, upregulator of β2 integrin affinity (3, 19). Indeed, PMo surface expression of β2 integrins, particularly αMβ2, is higher in the lung than in the blood, pointing to its tissue-dependent regulation (22, 49). Hyperlipidemia does not change the kinetic parameters of PMo except for the reduced speed, which correlates with a higher proportion of Lèvy-type tracks (21, 26) (**Table 1**). Lèvy-like walks are also visible in carotid arteries stimulated with a TLR7/8 agonist that induces endothelial cell damage (26). In this context, α4β1 integrin is required to resist shear stress (26), but since inhibition of the αLβ2 integrin reduces the number of PMo crawlers but not the frequency of Lèvy-type walks, αMβ2 integrin seems the main actor for this type of locomotion (26) (**Figure 1A**). Likewise, αMβ2 is necessary for longer interactions and above a shear stress threshold in contrast to αLβ2 integrin in neutrophils (50).

Nevertheless, the highest frequency of tracks resembling intermittent Lèvy-like motility is found in mouse vascular territories with deposits of harmful microparticles or aggregates including oxidized LDL (oxLDL) in the carotid artery of atheroprone mice fed a high-fat diet (21); β-amyloid aggregates in the lumen of brain veins in Alzheimer’s angiopathy (8, 51); apoptotic endothelial cells upon TLR7/8 stimulation in kidney glomerulus (2); circulating tumor metastasizing cells in lung capillaries (3); CYR61 secreted by platelets in mesenteric vessels after TLR7/8 addition (29); and sickle red blood cells (9, 52, 53), among others (**Figure 1A**). Notably, many of these particles can be recognized by αMβ2 integrin itself or in cooperation with other scavenger receptors such as CD36 and TLR (54, 55); in fact, CD36 or TLR7 deficiency decreased Lèvy-like PMo crawling on the vasculature of mice fed a high-fat diet or stimulated with a TLR7/8 agonist, respectively (2, 21). αMβ2 integrin is a promiscuous receptor that can bind more than 50 ligands including β-amyloid, iC3b, and CYR61 (56, 57). Although αMβ2 integrin binds ICAM-1 with lower affinity than αLβ2 integrin, it binds other ligands such as fibrinogen with 25-fold more affinity, which, together with their counter-regulated expression by inflammatory cytokines as TNFα (27), may confer αMβ2 integrin an advantage for endothelial interaction in the presence of deposits or damage. Indeed, blockade of αMβ2 integrin eradicates Lèvy-like tracks in for example steady-state mesenteric veins similarly to blockade of its ligand CYR61 after TLR7/8 stimulation (29). We propose to consider αMβ2 integrin as the essential damage receptor in PMo (beyond its function as an adhesion receptor) that seems to govern the Lèvy-like walk, acting as a decision-making receptor to integrate intravascular search and motility in homeostasis and pathology.

Outside-in signals by multivalence interaction may favor microparticle-induced clustering of αMβ2 integrin and thus its higher avidity (40) (**Figure 1A**). It will be interesting to explore whether αMβ2 integrin can reside in preformed nanoclusters in GPI domains as shown for αLβ2 integrin in monocytes and serve for its dynamic recruitment at adhesion sites for amoeboid locomotion (58). Indeed, PMo engulfing oxLDL *via* CD36 increased their levels of F-actin and upregulated genes related to Rab GTPases, integrin recycling, and lamellipodia formation (21), suggesting that this machinery may contribute to actin-driven amoeboid PMo motility during Lèvy-like walks in line with the role of actin flows in coupling speed and directional persistence (59). Intrinsic factors identified for intermittent (Lèvy-like) tissue motility in T cells include the unconventional myosin MYO1G that acts as a turning motor (35) and the Rho-associated protein kinase (ROCK) required for high-speed and directionality (30). Whether similar intrinsic regulators of speed fluctuations and turning patterns exist in PMo remains unknown.

αMβ2 integrin activity can also be regulated in circulating PMo by its crosstalk with other scavenger receptors as CD36 (**Figure 1A**), able of recognizing a variety of damage signals ranging from apoptotic cells to modified lipids (55). In a mouse model of atherosclerosis, CD36 uptake of oxLDL in PMo induces DAP12/Src family kinase (SFK) signaling and leads to increased F-actin polymerization (and probably higher β2 integrin avidity) and enhanced PMo Lèvy-like crawling (21), and a similar boosting of PMo particle engulfment is observed in sickle red blood cell clearance (9, 52, 53). This intravascular educational program constitutes an interesting feedforward mechanism for more efficient search and removal of particles by PMo, thus helping to prevent spread of inflammatory damage to the tissues.

Extrinsic factors as shear stress may regulate β2 integrin affinity (60) and ICAM-1 clustering (46), alter endothelial glycocalyx (61), and favor endothelial cell damage (26) or deposition of oxLDL (62) in areas of disturbed blood flow. The deposited microparticles can themselves promote local changes in the apical endothelial membrane that can augment the frequency of PMo Lèvy-like walks, such as increased membrane stiffness by the uptake of oxLDL by endothelial CD36 (62) and the pathogen-induced protrusion of microvilli (63), by altering specific lipid domains in both cases.

After meticulous amoeboid crawling on the endothelium during the slow phase of Lèvy-like walk, PMo become rounder and move quickly and directionally (flight) to the next area for another meticulous search (8) using mechanisms yet to be clarified. Several factors can underlie speed fluctuations (23), but although αLβ2 integrin/ICAM-1 interactions support high-speed and straight migration of T cells in the lymph node (64), movies of PMo Lèvy-like crawling show that this acceleration step seems to relate to PMo decreased adhesiveness (**Figure 1A**). Similar flights are perceived during PMo crawling in mice deficient in kindlin-3 or treated with SFK inhibitors pointing to reduced β2 integrin affinity as permissive for PMo high-speed step (2, 21, 22). PMo sliding behavior is also visible in CX3CR1-deficient mice (3, 22). Thus, although CX3CR1-CX3CL1 axis modulates Lèvy-like walks by regulating β2 integrin affinity (2, 3), it seems dispensable for the acceleration phase, and its absence does not seem to decrease the frequency of these tracks (1, 26, 29). The low-adhesive contact of PMo with the endothelium points to the involvement of low affinity and reversible receptor-ligand pairs resembling selectin interactions during rolling (4), although in the movies PMo seem to jump or slide rather than roll (8, 26). Since PMo do not express selectins, PSGL1 is a candidate to underlie PMo flights by its interaction with P-selectin expressed by the endothelium under certain stimulation and regulated by preformed membrane microdomains (65) (**Figure 1A**). The carbohydrate modification of PSGL-1 Slan is a marker for a subset of PMo and modulates innate and adaptive immune responses, but its possible contribution to PMo crawling has not been investigated (66, 67).

Therefore, the local presence of microparticles or damage on endothelial cells are the key factors to promote environmental-guided Lèvy-like PMo migration. This fact may explain the lack of Lèvy-like tracks *in vitro* since although inflammatory cytokines and flow were incorporated, no aggregates were present (25). Moreover, if there is massive endothelial damage or larger deposits, Lèvy-like walk will change into confined meticulous crawling (23) as observed in the lung 1 day after injection of tumor cells (3) and near arterial atheroma plaques (26) (**Table 1**), allowing enough time for PMo interaction to increase the likelihood of engulfment. Increased retention during Lèvy-like or confined crawling due to the geometric constraints of certain vascular territories could also induce the production of chemokines and cytokines by PMo and/or the endothelial cells with which they interact. These soluble factors will serve to recruit cooperating circulating leukocytes such as neutrophils to cope with dying endothelial cells in response to TLR7/8 stimulation in the glomerulus (2) and natural killer cells to help eliminate circulating tumor cells in the lung microvasculature (3).

### Post-Crawling Detachment

After crawling PMo usually undergo detachment, a key step to maintain PMo surveying the vasculature by avoiding their extravasation. This allows several rounds of endothelial scanning by PMo and prevents spreading of damaging microparticles to the tissues. Our group recently identified that the GPI-anchored protease MT4-MMP could cleave the αM integrin chain at N^977^L position (not conserved in αL integrin) serving as a possible mechanism for PMo post-crawling detachment (12) (**Figure 1B**). Accordingly, in MT4-MMP absence there were increased numbers of PMo crawling on the activated endothelium of the cremaster muscle in an αMβ2 integrin-dependent manner and also transmigrating into the inflamed aorta (12). These data support that PMo detachment post-crawling prevents β2 integrin-dependent transendothelial migration (27). These findings also indicate that a pool of αMβ2 integrin molecules reside at GPI microdomains of the PMo plasma membrane what could account for fine-tuned contribution of αMβ2 integrin to diffusive random or Lèvy-like walk crawling, and in particular to PMo post-crawling detachment. Of interest, shear stress can induce αM integrin cleavage by cathepsin B in neutrophils (68). Whether this cleavage, β2 integrin processing by other proteases (69, 70), or proteolysis-independent mechanisms play additional roles in PMo detachment post-crawling remains unknown.

## Conclusions and Perspectives

Leaving aside the discussion about true Lèvy-like walks in biological systems (24), we consider interesting to complement the current perspective of intravascular PMo crawling with the point of view that the search mode influences PMo motility as proposed for tissue T cells (23). Although PMo motility patterns are far more complex than the simplification herein proposed, this change of paradigm may help understand PMo crawling better and identify novel regulators to boost PMo protective intravascular actions and prevent disease. Open questions remain about the dynamic regulation of integrins and other intrinsic actors (actin, GTPases), endothelial players, and extrinsic factors (shear stress) during these distinct modes of PMo crawling. It would also be necessary to determine instantaneous and individual track PMo kinetic parameters in future work.

These questions undoubtedly raise the need to implement innovative techniques and tools to fully understand these events at the single-cell scale and *in vivo*. For example: (i) novel *in vitro* settings for live time-lapse to recapitulate *in vivo* complexity using immobilized ligands in lipid bilayers and under flow (46); (ii) advanced microscopy techniques for visualization and 3D reconstruction of intravascular PMo (71) together with novel PMo markers as PD-L1 (34) to avoid the limitation of CX3CR1 heterozygous mice; and (iii) innovative techniques for *in vivo* single-molecule tracking to characterize receptor and ligand clustering at PMo and endothelial plasma membrane (29, 72, 73).

## Author Contributions

RM-C and LL-M prepared the Table and Figure, respectively. RM-C, LL-M, and AGA wrote the text. All authors contributed to the article and approved the submitted version.

## Funding

The work in this manuscript has been funded by the Spanish Ministry of Science and Innovation (grants SAF2017-83229-R and PID2020-112981RB-I00 to AGA and fellowship PRE2018-085163 to LL-M). RM-C has a contract with the Community of Madrid co-financed by the European Social Fund for Youth Employment. We acknowledge support of the publication fee by the CSIC Open Access Publication Support Initiative through its Unit of Information Resources for Research (URICI).

## Conflict of Interest

The authors declare that the research was conducted in the absence of any commercial or financial relationships that could be construed as a potential conflict of interest.

## Publisher’s Note

All claims expressed in this article are solely those of the authors and do not necessarily represent those of their affiliated organizations, or those of the publisher, the editors and the reviewers. Any product that may be evaluated in this article, or claim that may be made by its manufacturer, is not guaranteed or endorsed by the publisher.
